# Risk of cancer in a large cohort of nonaspirin NSAID users: a population-based study

**DOI:** 10.1038/sj.bjc.6600945

**Published:** 2003-05-27

**Authors:** H T Sørensen, S Friis, B Nørgård, L Mellemkjær, W J Blot, J K McLaughlin, A Ekbom, J A Baron

**Affiliations:** 1 Department of Clinical Epidemiology, Aarhus University and Aalborg Hospital, Vennelyst Boulevard 6, Building 260, DK-8000 Aarhus C, Denmark; 2 Department of Medicine, Vanderbilt University Medical Center and the Vanderbilt-Ingram Cancer Center, Nashville, TN, USA; 3 Institute of Cancer Epidemiology, The Danish Cancer Society, Strandboulevarden 49, DK-2100 Copenhagen Ø, Denmark; 4 International Epidemiology Institute, Ltd, 1455 Research Blvd., Suite 550, Rockville, MD 20850, USA; 5 Unit of Clinical Epidemiology, Department of Medicine, Karolinska Hospital, Box 281, SE-171 77 Stockholm, Sweden; 6 Departments of Medicine and Community and Family Medicine, Dartmouth Medical School, Hanover, NH 03756, USA

**Keywords:** NSAIDs, epidemiology, risk, prevention

## Abstract

There is increasing evidence of an inverse association between use of nonsteroidal anti-inflammatory drugs (NSAIDs) and risk of colorectal cancer. However, data regarding other cancer sites are limited. Using data from the population-based North Jutland Prescription Database and the Danish Cancer Registry, we compared cancer incidence among 172 057 individuals prescribed nonaspirin NSAIDs with expected incidence (based on county-specific cancer rates) during a 9-year study period. A total of 6081 incident cancer cases were diagnosed among NSAID users *vs* 5722 expected (standardised incidence ratio (SIR) 1.1, 95% confidence interval (CI)1.0–1.1). The SIRs for colon and rectal cancer among persons who obtained 10 or more prescriptions were 0.7 (95% CI 0.6–0.9) and 0.6 (95% CI 0.4–0.9), respectively. Similarly, reduced risk estimates were found for stomach (SIR 0.7, 95% CI 0.4–1.1) and ovarian cancer (SIR 0.7, 95% CI 0.4–1.0). Standardised incidence ratios for other cancers among those with 10 or more prescriptions tended to be close to 1.0, except for lung, kidney, and prostate cancers with SIRs of 1.3 (95% CI 1.1–1.6), 1.4 (95% CI 0.9–2.1), and 1.6 (95% CI 1.3–2.0), respectively. We found protective associations of NSAIDs against colon, rectal, stomach, and ovarian cancer. Reasons for the increased risk for some cancer sites are not clear.

There is substantial evidence of an inverse association between use of nonsteroidal anti-inflammatory drugs (NSAIDs) and the risk of colorectal cancer (Kune *et al*, 1988; Rosenberg *et al*, 1991,^1998^; Thun *et al*, 1991,^1993^; Suh *et al*, 1993; Muscat *et al*, 1994; Peleg *et al*, 1994; Schreinemachers *et al*, 1994; Giovannucci *et al*, 1995; Smalley *et al*, 1999; Baron and Sandler, 2000; Coogan *et al*, 2000; Langman *et al*, 2000; Rodrigues and Huerta-Alvarez, 2001). A protective effect was initially observed in experimental studies and has been seen in epidemiologic studies of both cohort and case–control designs. The mechanisms underlying any chemopreventive effect of NSAIDs are not clear, but inhibition of cyclooxygenase 2 (COX-2) is a prominent candidate (DuBois and Smalley, 1996; Kawamori *et al*, 1998; Jones *et al*, 1999; Morris *et al*, 2001).

There have been relatively few studies of possible effects of NSAID use on the risk of other cancers, although protective effects for oesophageal and stomach cancers have been reported (Thun *et al*, 1993; Funkhouser and Sharp, 1995; Garidou *et al*, 1996; Farrow *et al*, 1998). In contrast, a recent study reported an increased risk of pancreatic and prostate cancer among NSAID users (Langman *et al*, 2000). Data regarding the risk of lung, breast, and ovarian cancer among NSAID users are conflicting (Peto *et al*, 1988; Paganini-Hill *et al*, 1989; Schreinemachers and Everson, 1994; Egan *et al*, 1996; Harris *et al*, 1996,^1999^; Cramer *et al*, 1998; Akhmedkhanov *et al*, 2001; Moysich *et al*, 2001; Meier *et al*, 2002).

We have therefore linked the Pharmaco-Epidemiological Prescription Database in North Jutland County and the Danish Cancer Registry to examine cancer incidence in a large population-based cohort of NSAID users.

## METHODS

The study was carried out within the population of North Jutland, a Danish county with nearly 500 000 inhabitants. The details of the study design have been described earlier (Friis *et al*, 2003). The county is served by pharmacies equipped with a computerised accounting system from which data are sent to the Danish National Health Service, which refunds patients part of the costs associated with the purchase of drugs, including NSAIDs, prescribed by doctors, and the prescription data are transferred to the Pharmaco-Epidemiological Prescription Database (Gaist *et al*, 1997; Sørensen *et al*, 2000), including the customer's unique civil registry number, the type of drug prescribed (Gaist *et al*, 1997; Sørensen *et al*, 2000), and the date of prescription. The unique civil registry number ensures that complete individual prescription histories can be established.

The Prescription Database was used to identify NSAID prescriptions for 190 753 county residents between 1 January 1989 and 31 December 1995. The identified prescriptions were for the following NSAIDs: azapropazone, diclofenac, etodolac, fenbufen, fenoprofen, flubiprofen, ibuprofen, indomethacin, ketoprofen, ketorolac, nabumetone, naproxen, phenylbutazone, piroxicam, proquazon, sulindac, tenoxicam, tiaprofenic acid, tolfenamic acid, and tolmetin. In Denmark, all NSAIDs can be obtained only by prescription, except for aspirin and low doses of ibuprofen.

Overall, 7060 (3.7%) of the identified persons prescribed NSAIDs were excluded from the study cohort because of (i) residency outside the county of North Jutland at the date of prescription (*n*=6272); (ii) invalid civil registry number (*n*=54); (iii) death prior to or at the date of prescription (*n*=55); (iv) parent (of patient) registered as customer (*n*=574); or (v) age below 16 years (*n*=105). After these exclusions, 183 693 (96.3%) persons were left for subsequent record linkage.

The study cohort was linked to the files of the Danish Cancer Registry and subjects with a cancer diagnosis, except nonmelanoma skin cancer, prior to the date of first recorded prescription for NSAID (*n*=7403; 3.9%) were excluded from the analyses. The follow-up period began 1 year after the date of the first recorded NSAID prescription and ended on the date of first primary cancer diagnosis (except nonmelanoma skin cancer), date of death, emigration, or 31 December 1997, whichever occurred first. Data on death for subjects who died during follow-up were obtained through linkage to the National Mortality Files. To reduce any bias introduced by the inclusion of patients with recent or undiagnosed cancer, we excluded the person-time and cancer experience in the first year of follow-up after the first NSAID prescription, involving 4233 persons (2.2%) who had a cancer diagnosis (*n*=1597) or died (*n*=2636) within the first year of follow-up. After these exclusions, the study cohort included 172 057 (90.2%) individuals.

The number of cancer cases observed among users of NSAIDs was compared with the number expected, based on county-specific cancer incidence rates from the Danish Cancer Registry. Expected numbers of first primary cancers in the study cohort were calculated by multiplying the number of person-years of the cohort members (in sex groups and 5-year age groups) by the corresponding 5-year age group and calendar-year-specific incidence rates of first primary cancers for all inhabitants of North Jutland county who had not received a prescription for an NSAID. The standardised incidence ratio (SIR) was calculated as the ratio of the observed to the expected number of cancer cases. We also computed SIRs stratified by number of prescriptions for NSAIDs. For these analyses, the person-time experience of the study subjects was distributed among four categories of prescription frequency (1; 2–4; 5–9; or ⩾10 prescriptions) with follow-up for cancer beginning on the date of the first prescription within the given category. We also performed a test for trend in SIRs with the number of prescriptions. The statistical methods employed assume that the observed number of cases of cancer in any specific category followed a Poisson distribution. We calculated 95% confidence intervals (CI) for the SIR from an accurate asymptotic approximation.

## RESULTS

Table 1

**Table 1:** Characteristics of 172 057 users recorded in the Prescription Database of North Jutland County, Denmark, between 1 January 1989 and 31 December 1995

	**Number of users**
*Sex*	
Men	78.562
Women	93.495
*Age at entry*^a^ (years)	
<50	99.376
50–69	46.714
⩾70	25.967
*Year of entry*^a^	
1989	27.258
1990–1991	51.053
1992–1993	49.08
1994–1995	44.666
*Number of prescriptions*	
1	71.603
2–4	59.964
5–9	21.398
⩾10	19.092

a Date of first recorded prescription.

shows characteristics of the study cohort of 172 057 persons free of cancer at the start of follow-up. The mean age at entry in the study, that is, time of the first recorded prescription for NSAIDs, was 47.2 years (standard deviation 18.6), and the mean follow-up after first prescription was 5.4 years (standard deviation 2.1, range 1–9 years), generating 751 182 person-years. In all, 58% of the study subjects received two or more prescriptions for NSAIDs during the registration period, with 11% receiving 10 or more prescriptions.

Overall, 6081 incident cancer cases were diagnosed among NSAID users *vs* 5722 expected, yielding an SIR of 1.1 (95% CI: 1.0–1.1) (Table 2

**Table 2:** Standardised incidence ratios (SIRs) and 95% confidence intervals (CI) for cancers of selected sites in users of NSAIDs in North Jutland, Denmark

**Cancer site (modified ICD-7 code)**	**Observed**	**SIR**	**95% CI**
*All neoplasms*	6081	1.1	1.0–1.1
Men	2709	1.1	1.0–1.1
Women	3372	1.1	1.0–1.1
			
Buccal cavity (140–148)	98	0.9	0.7–1.0
Oesophagus (150)	43	0.8	0.6–1.1
Stomach (151)	131	0.9	0.8–1.1
Colon (153)	427	0.9	0.8–1.0
Rectum (154)	221	0.9	0.7–1.0
Liver (155)	57	1.4	1.0–1.8
Pancreas (157)	149	1.1	0.9–1.2
Lung, primary (162)	692	1.1	1.0–1.2
Breast (170)	696	1.1	1.0–1.2
Ovary (175)	130	0.9	0.7–1.0
Cervix uteri (179)	72	0.6	0.5–0.8
Corpus uteri (172)	148	1.1	0.9–1.3
Prostate (177)	324	1.3	1.2–1.5
Testis (178)	45	1.0	0.7–1.4
Kidney (180)	144	1.2	1.0–1.5
Urinary bladder (181)	330	1.2	1.0–1.3
Melanoma (190)	167	1.0	0.8–1.1
Nonmelanoma skin cancer (191)	1093	1.1	1.0–1.2
Brain (193)	170	1.2	1.0–1.3
Non-Hodgkin's lymphoma (200, 202)	148	1.1	0.9–1.2
Hodgkin's disease (201)	23	1.5	0.9–2.2
Multiple myeloma (203)	78	1.6	1.2–2.0
Leukaemia (204)	123	0.9	0.7–1.1

). Among the 23 specific types of cancer listed in the table, the SIRs for all, but five, fell between 0.8 and 1.2. For gastrointestinal cancers, the SIRs among NSAID users were 0.9 (95% CI 0.8–1.0) for colon cancer, 0.9 (95% CI 0.7–1.0) for rectum, 0.8 (95% CI 0.6–1.1) for oesophagus, 0.9 (95% CI 0.8–1.1) for stomach, and 1.1 (95% CI 0.9–1.2) for pancreas cancer. Increased SIRs were found for cancers of the prostate (SIR, 1.3; 95% CI 1.2–1.5), kidney (SIR 1.2; (95% CI 1.0–1.5), and for multiple myeloma (SIR, 1.6; 95% CI 1.2–2.0), while a reduced risk of cervical cancer was seen (SIR, 0.6; 95% CI 0.5–0.8). The results for site-specific cancers were comparable in men and women (data not shown).

Table 3

**Table 3:** Standardised incidence ratios (SIRs) and 95% confidence intervals (CI) for cancers of the gastrointestinal tract including pancreas, stratified by number of NSAID prescriptions

**Cancer site**		**Observed**	SIR	95% CI
*Oesophagus*				
Number of prescriptions	1	10	0.6	0.3–1.1
	2–4	15	0.9	0.5–1.4
	5–9	8	0.9	0.4–1.9
	⩾10	10	1.1	0.5–2.0
Test for trend				*P*=0.21
				
*Stomach*				
Number of prescriptions	1	35	0.8	0.6–1.1
	2–4	58	1.2	0.9–1.6
	5–9	20	0.9	0.5–1.3
	⩾10	18	0.7	0.4–1.1
Test for trend				*P*=0.54
				
*Colon*				
Number of prescriptions	1	130	0.9	0.8–1.1
	2–4	164	1.0	0.9–1.2
	5–9	66	0.8	0.6–1.0
	⩾10	67	0.7	0.6–0.9
Test for trend				*P*=0.05
				
*Rectum*				
Number of prescriptions	1	67	0.8	0.6–1.0
	2–4	86	1.0	0.8–1.2
	5–9	38	0.9	0.6–1.2
	⩾10	30	0.6	0.4–0.9
Test for trend				*P*=0.34
				
*Pancreas*				
Number of prescriptions	1	30	0.7	0.5–1.0
	2–4	58	1.2	0.9–1.6
	5–9	37	1.6	1.1–2.2
	⩾10	24	0.9	0.6–1.3
Test for trend				*P*=0.16

presents results stratified by number of prescriptions for cancers of the gastrointestinal tract, the sites of *a priori* interest. For colon, rectum, and stomach cancers, the lowest SIRs (0.7, (95% CI 0.6–0.9); 0.6, (95% CI 0.4–0.9) and 0.7, (95% CI 0.4–1.1), respectively) were found among persons who obtained 10 or more prescriptions.

Results of similar analyses for other cancer sites are presented in Table 4

**Table 4:** Standardised incidence ratios (SIR) and 95% confidence intervals (CI) for selected nongastrointestinal cancers stratified by number of NSAID prescriptions

Characteristics		Observed	SIR	95% CI
*Lung, primary*				
Number of prescriptions	1	177	0.9	0.8–1.0
	2–4	241	1.1	1.0–1.3
	5–9	135	1.4	1.1–1.6
	⩾10	139	1.3	1.1–1.6
Test for trend				*P*=0.0005
				
*Breast*				
Number of prescriptions	1	193	1.0	0.8–1.1
	2–4	258	1.1	1.0–1.3
	5–9	120	1.1	0.9–1.3
	⩾10	125	1.1	0.9–1.3
Test for trend				*P*=0.21
				
*Cervix*				
Number of prescriptions	1	24	0.6	0.4–0.8
	2–4	24	0.6	0.4–0.8
	5–9	10	0.6	0.3–1.1
	⩾10	14	0.9	0.5–1.5
Test for trend				*P*=0.24
				
*Ovary*				
Number of prescriptions	1	34	0.7	0.5–1.0
	2–4	53	1.0	0.8–1.3
	5–9	26	1.1	0.7–1.5
	⩾10	17	0.7	0.4–1.0
Test for trend				*P*=0.99
				
*Prostate*				
Number of prescriptions	1	87	1.1	0.9–1.4
	2–4	105	1.3	1.0–1.5
	5–9	61	1.5	1.1–1.9
	⩾10	71	1.6	1.3–2.0
Test for trend				*P*=0.02
				
*Bladder*				
Number of prescriptions	1	102	1.1	0.9–1.3
	2–4	116	1.2	1.0–1.4
	5–9	51	1.1	0.8–1.4
	⩾10	61	1.2	0.9–1.6
Test for trend				*P*=0.58
				
*Kidney*				
Number of prescriptions	1	37	1.0	0.7–1.4
	2–4	46	1.1	0.8–1.5
	5–9	33	1.8	1.2–2.5
	⩾10	28	1.4	0.9–2.1
Test for trend				*P*=0.04
				
*Multiple myeloma*				
Number of prescriptions	1	13	0.9	0.5–1.5
	2–4	35	2.1	1.4–2.9
	5–9	19	2.3	1.4–3.6
	⩾10	11	1.2	0.6–2.2
Test for trend				*P*=0.28

. The SIRs for prostate cancer increased with increasing numbers of prescriptions, with an SIR of 1.6 (95% CI 1.3–2.0) among those with 10 or more prescriptions. Among persons who obtained 10 or more prescriptions, the SIR for cancer of the ovary was decreased (SIR 0.7, 95% CI 0.4–1.0), while for cervical cancer, there was no evidence of decreasing SIRs with increasing prescriptions, and for breast cancer the risk estimates were all close to unity. For lung, bladder, and kidney cancer, we found SIRs above 1.0 in almost all categories of prescription frequency, with significant trends for all but bladder cancer. Among persons who obtained two to four or five to nine prescriptions, the SIRs for multiple myeloma were increased, although without any obvious trend with increasing number of prescriptions.

## DISCUSSION

In this large population-based follow-up study, we found significantly reduced risks of colon and rectal cancers, and a trend towards a reduced risk of stomach cancer among users of nonaspirin NSAIDs who had filled 10 or more prescriptions. Overall, we found moderate increased risks of cancers of the prostate and kidney. We did not confirm a reduced risk of lung or breast cancer as reported in some studies (Schreinemachers and Everson, 1994; [Bibr bib15][Bibr bib16]; Akhmedkhanov *et al*, 2002). Finally, our data support a reduced risk for ovarian cancer, which has also been reported among both users of paracetamol and aspirin (Cramer *et al*, 1998; Akhmedkhanov *et al*, 2001), and we observed a reduced risk of cervical cancer, although not among those with 10 or more prescriptions. To our knowledge, this association has not been previously investigated, although an increase of COX-2 expression has been reported in cervical cancer (Kulkarni *et al*, 2001; Sales *et al*, 2001,^2002^).

With few exceptions, studies with different designs and populations have shown that aspirin and other NSAIDs appear to decrease the risk of colorectal cancer by up to 50% (Baron and Sandler, 2000). The consistency of these findings and the biological mechanisms that appear to explain them (DuBois and Smalley, 1996) suggest that the association is likely to be causal. A similar protective effect has not been found for paracetamol, further indicating that the reduced risk is not because of an effect of the underlying disease (Baron and Sandler, 2000). Some studies suggest that up to 10–20 years of regular use of NSAIDs is required before a substantially decreased risk of colorectal cancer can be detected (Thun *et al*, 1993; Giovannucci *et al*, 1995; Baron and Sandler, 2000), although others have shown lower rates of colorectal cancer within 5 years of NSAID use (Smalley *et al*, 1999; Rodrigues and Huerta-Alvarez, 2001). An early stage effect appears likely, especially since aspirin has been associated with a clearly reduced risk of large-bowel adenomas that are considered a precursor of most colorectal cancers (Greenberg *et al*, 1993; Sandler *et al*, 1998). However, probably there is a threshold for the needed dose, since several studies have shown that low-dose aspirin does not have a major cancer protective effect (Friis *et al*, 2003).

Our data also add to the literature suggesting that NSAIDs may exert an antineoplastic effect in the stomach. However, unlike some previous investigations (Funkhouser and Sharp, 1995; Garidou *et al*, 1996; Farrow *et al*, 1998), we saw no substantial indication that NSAID use is inversely related to risk of cancer of the oesophagus.

Previous studies have not indicated a consistent association of NSAIDs with haematopoietic cancer or cancers of the urinary or genital tract (Kune *et al*, 1988; Paganini-Hill *et al*, 1989; Thun *et al*, 1993; Baron and Sandler, 2000). Our findings of elevated risks for cancers of the prostate, kidney, and multiple myeloma among users of only few prescriptions may indicate that NSAIDs were prescribed for alleviation of early symptoms of undiagnosed cancer. Even though we excluded the first year of follow-up, we may not have avoided this possible confounding by indication, as some of these cancers may present with long periods with uncharacteristic symptoms before diagnosis. In addition, since screening for prostate cancer has increased during the last decades, surveillance bias may have contributed to the elevated relative risk estimates for this cancer. One recent study has suggested that daily use of NSAIDs may be associated with a lower incidence of prostate cancer (Roberts *et al*, 2002) in contrast to our findings and data reported from Great Britain (Langman *et al*, 2000).

Only phenacetin-containing drugs have been causally linked to renal cancer (McLaughlin and Lipworth, 2000), but our data cannot exclude that an association with other NSAIDs may exist. Since we did not find any strong association between NSAID use and lung cancer or other smoking-related cancers, it is unlikely that cigarette smoking is responsible for the observed associations with kidney cancer (McLaughlin and Lipworth, 2000). Obesity could be a confounding factor for the latter malignancy, since it is strongly related to both kidney cancer and osteoarthritis, a common indication for NSAIDs.

Some studies have examined in detail the risk of lung cancer among NSAID (primarily aspirin) users. Two follow-up studies did not find any overall association with aspirin use, but there seemed to be an inverse association with lung cancer incidence (Paganini-Hill *et al*, 1989) and mortality among women (Thun *et al*, 1993). Two case–control studies (Rosenberg, 1995; Langman *et al*, 2000) reported no association between NSAIDs and lung cancer, but a third found a relative risk of 0.7 (95% CI 0.3–1.3) among women taking aspirin for at least 6 months (Akhmedkhanov *et al*, 2002). We had no information on cigarette smoking in our population, and thus we cannot rule out that the small SIR elevations for lung cancer (similar in men and women) may be because of differential smoking rates among NSAID users.

The main strengths of our study are its large size, its population-based design, the completeness of follow-up, and the high quality of the cancer registration (Storm *et al*, 1997). The use of a prescription database eliminates recall bias, which may distort findings in case–control studies. Unfortunately, we do not have information on drug use prior to 1989, but large numbers of users with repeat prescriptions suggest that many subjects were prevalent users. We also lack clinical details about the indications for NSAID use and underlying diseases. Another limitation is the relatively short follow-up period, since long-term use may be required before a reduced risk of cancer appears (Thun *et al*, 1993; Giovannucci *et al*, 1995; Baron and Sandler, 2000). From another study, with the aim to study the risk of gastrointestinal bleeding, it seems that a prescription lasts 60–90 days (Mellemkjaer *et al*, 2002). In addition, we were unable to control for smoking, dietary habits, alcohol intake, and other factors. Furthermore, we had no data on compliance with the prescriptions or use of over-the-counter NSAIDs. However, the fact that drug exposure was based on prescriptions actually dispensed at pharmacies and paid in part by the patients is likely to have improved compliance. In general, over-the-counter use of nonaspirin NSAIDs in Denmark is 14% of the total NSAID use (Mellemkjær *et al*, 2002), but over-the-counter use may be less common among persons with prescription use, so possible confounding by over-the-counter use would lead to underestimation of a reduced cancer risk associated with NSAIDs. Whatever the impact of these issues, the fact that our estimates for the effect of NSAID use on risk of colorectal cancer agree closely with previous findings tends to support the general validity of our approach.

Our study findings provide further support that NSAIDs may protect against colorectal and ovarian cancers and perhaps stomach cancer, in particular, after 10 prescriptions. The increased risk ratios observed for other cancers need to be investigated further.
